# Do placebo expectations influence perceived exertion during physical exercise?

**DOI:** 10.1371/journal.pone.0180434

**Published:** 2017-06-29

**Authors:** Hendrik Mothes, Christian Leukel, Harald Seelig, Reinhard Fuchs

**Affiliations:** 1 Department of Sports Science, University of Freiburg, Schwarzwaldstrasse 175, Freiburg, Germany; 2 Bernstein Center Freiburg, University of Freiburg, Hansastrasse 9a, Freiburg, Germany; 3 Department of Sport, Exercise and Health, University of Basel, Birsstrasse 320 B, Basel, Switzerland; Sao Paulo State University, BRAZIL

## Abstract

This study investigates the role of placebo expectations in individuals’ perception of exertion during acute physical exercise. Building upon findings from placebo and marketing research, we examined how perceived exertion is affected by expectations regarding a) the effects of exercise and b) the effects of the exercise product worn during the exercise. We also investigated whether these effects are moderated by physical self-concept. Seventy-eight participants conducted a moderate 30 min cycling exercise on an ergometer, with perceived exertion (RPE) measured every 5 minutes. Beforehand, each participant was randomly assigned to 1 of 4 conditions and watched a corresponding film clip presenting “scientific evidence” that the exercise would or would not result in health benefits and that the exercise product they were wearing (compression garment) would additionally enhance exercise benefits or would only be worn for control purposes. Participants’ physical self-concept was assessed via questionnaire. Results partially demonstrated that participants with more positive expectations experienced reduced perceived exertion during the exercise. Furthermore, our results indicate a moderator effect of physical self-concept: Individuals with a high physical self-concept benefited (in terms of reduced perceived exertion levels) in particular from an induction of generally positive expectations. In contrast, individuals with a low physical self-concept benefited when positive expectations were related to the exercise product they were wearing. In sum, these results suggest that placebo expectations may be a further, previously neglected class of psychological factors that influence the perception of exertion.

## Introduction

The rate of perceived exertion measures how arduous someone perceives a particular physical exercise as being. Previous studies [1–3] propose that the degree to which people perceive their exercise as being arduous negatively influences how they respond affectively during the exercise. Negative affective responses, in turn, decrease long-term exercise motivation and participation [1]. If one wishes to counteract the detrimental effects of a sedentary lifestyle, it is thus important to understand factors that affect perceived exertion in exercising individuals.

While the experience of exertion clearly depends on exercise intensity [4] and corresponding physiological cues (e.g., ventilation, oxygen uptake, skin temperature) [5], it also involves psychological factors [6]. For instance, it has been shown that dispositional factors, such as anxiety, neuroticism, or self-efficacy, and situational factors, such as specific mood states (anxiety, depression), may influence perceived exertion [7,8]. Nevertheless, psychological influences on the perception of exertion are poorly understood so far. We propose in the present study that placebo-like beliefs and expectations may be a further, previously neglected yet powerful class of psychological factors influencing perceived exertion.

In medical placebo research, expectations that a certain behavior (e.g., taking a pill) will have positive or negative effects on health status (outcome expectations) are considered a key factor shaping not only various long-term diseases, but also certain underlying physical or mental processes [9–11]. In particular, substantial research demonstrates that expectations influence the detection of somatic sensations [12], a process that also plays a fundamental role in the perception of exertion during physical exercise [4]. In addition, previous research has frequently linked the effects of placebo expectations to self-perception [13,14]. Individuals with positive self-related cognitions derive particular benefit from placebo expectations [15,16]. Recent evidence shows that individuals with a negative self-concept can also benefit from placebo expectations if they are associated with an external product that supposedly supports the individual [17].

In the present study, we transferred current knowledge from placebo research to short-term (single session) physical exercise. We examined whether subtle manipulations of participants’ expectations (*induced expectations*) towards exercise-related health outcomes and the exercise product they were wearing might influence their perceived exertion. We expected that participants with positive induced expectations would experience less perceived exertion than participants without positive induced expectations. Further, we expected that physical self-concept would have a moderating effect [18]: People with a higher physical self-concept should benefit more (decreased perceived exertion) from positive outcome expectations [15,16]. In contrast, participants with a low physical self-concept should benefit particularly from positive outcome expectations when they are related to an external supportive product [17].

## Materials and methods

### Participants

**Fig 1:**
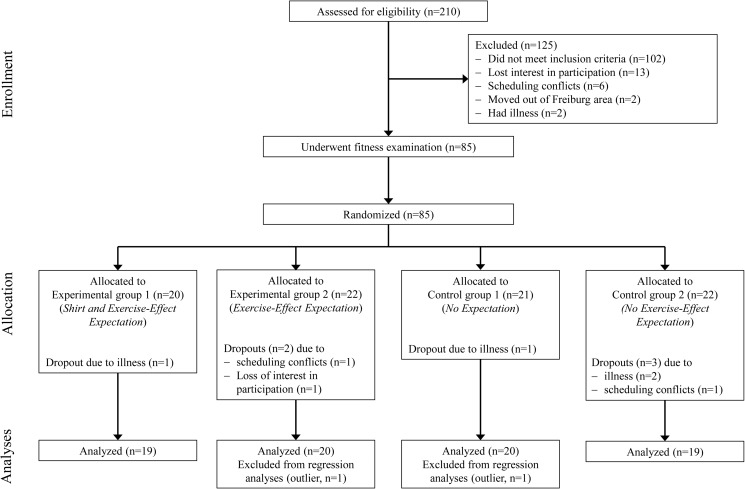
Participant flow diagram.

We recruited 210 potential participants via advertisements in local newspapers and at two local universities (University of Freiburg and Freiburg University of Education, Germany) from November 2012 to February 2013. Following recruitment, we prescreened all potential participants during a telephone interview. For further participation in the study, we invited healthy individuals who were older than 18 years and fluent in German and who reported neither substantial current levels of physical exercise (defined as regular physical activity of more than 60 min/week during the previous 3 months) nor substantial past levels of physical exercise (defined as involvement in sports clubs or competitions). We selected low-active to sedentary individuals, because we assumed these individuals to be the most sensitive to inductions of positive outcome expectations towards physical activity [19, 20]. To ensure sufficient naivety of participants, we selected only individuals who were not enrolled in psychology, medicine, biology, or sports science degree programs. Seventy-eight individuals (57 females, 21 males, *M*_age_ = 21.9, age range: 18–32 years) completed the study (see Fig 1 for the participant flow through the study). All participants gave their written informed consent, were debriefed after completion of the study, and were compensated for participation with 20 €. This study was part of a larger project examining acute psychological, physiological, and neurophysiological health-related effects of beliefs and expectations of single sessions of exercise [21]. It was approved by the Ethics Commission of the University of Freiburg and was in accordance with the latest revision of the Declaration of Helsinki.

### Design and procedure

We scheduled participants for two experimental sessions at the Department of Sports Science Laboratory at University of Freiburg, Germany. The two sessions were approximately one month apart (on average, 34 days). In the first session (lasting for 30 minutes), participants completed a set of questionnaires, which included scales for measuring their physical self-concept [18] and habitual expectation towards the affective consequences of exercising (as well as other scales not relevant for this study) [22]. Afterwards, we had the participants take a standardized fitness test on a bicycle ergometer (Ergo-Bike Medical8i_2, Daum, Germany) to measure their individual physical fitness. Following the fitness testing, participants were randomly assigned (stratified by gender) to one of four groups.

In the second session (main experiment, lasting 2 hours), participants were first instructed to put on a sleeveless compression shirt from a well-known sports brand to be worn during the experimental session. Participants then completed several questionnaires as well as blood pressure and electroencephalogram (EEG) measurements not relevant for the current analyses. After viewing a 3 min multimedia film clip (Induced Expectation), participants were instructed to warm up for two minutes (resistance in watts: 0.5 × body weight) and then to exercise with moderate intensity for 30 minutes on the bicycle ergometer while their rate of perceived exertion (RPE; [23,24]) was measured every five minutes. After finishing the exercise, participants again completed several questionnaires as well as blood pressure and EEG measurements not relevant for the present study. All study materials (instructions, measures, multimedia film clip) were provided in German to the participants and translated into English for this publication. The temperature in the laboratory was kept constant (20°C, 68°F) during all experimental sessions.

### Induced expectation

Participants were told that the purpose of the short multimedia film clip presented directly before the beginning of the physical exercise was to provide them more detailed information about the background of the study. Unknown to the participants, however, the film clip aimed to induce different placebo-like outcome expectations by presenting supposedly scientific information. According to his or her group assignment, each participant watched one of four 3-min multimedia presentations consisting of spoken words by a credible speaker with corresponding pictures (similar to those used in previous studies, e.g., [25]). Two conditions aimed at inducing positive outcome expectations (*Exercise-Effect Expectation* and *Shirt and Exercise-Effect Expectation*, referred to as Experimental 1 and Experimental 2). In both conditions, the film clips indicated that the specific duration and intensity of the exercise the participants were about to engage in was optimized to maximize mental health and well-being according to scientific evidence; the clips differed only with respect to the information about the compression shirt that all participants were wearing. Whereas the *Exercise-Effect Expectation* video (along with the control condition videos) informed them that the shirt’s function was to reduce body perspiration during the exercise for accurate EEG measurements, only the *Shirt and Exercise-Effect Expectation* video claimed that the compression shirt supported cardiovascular function and breathing during the exercise, thereby additionally increasing psychological well-being and the health effects of the exercise.

The two other conditions (*No Expectation* and *No Exercise-Effect Expectation*) served as control conditions. The *No Expectation* (referred to as Control 1) film clip was designed to not cause a particular outcome expectation regarding the upcoming exercise, as it only included information about the study that all participants had already received at the beginning of the experiment. Due to our concern that it would be difficult to rule out the influence of pre-existing positive expectations with the Control 1 condition, we introduced Control 2 as an additional control condition. We designed Control 2 as a *No Exercise-Effect Expectation*, that is, participants viewing the Control 2 film clip were not supposed to expect any immediate exercise benefit (“According to recent research, the upcoming exercise is too short and too weak to result in higher well-being and health benefits.”). This approach is in line with the hidden treatment paradigm frequently used in placebo research [26]. Moreover, none of the videos explicitly mentioned exertion during the exercise. Instead of creating exertion-specific expectations, the expectation manipulations aimed to induce global expectations regarding the consequences of the exercise. The reason for this was that the larger study aim was to investigate the effects of beliefs and expectations on acute exercise consequences (see also [21]). Nonetheless, there is also research indicating that vague information can be more beneficial than precise information in the process of expectation generation, because vagueness allows people to interpret the information the way they want [27]. We retrospectively double-checked via questionnaire whether our manipulation had successfully influenced the participants’ exercise experience. As our expectation manipulation was specifically focused on exercise duration and intensity, we assessed individual ratings of comfort with respect to duration and intensity in particular. Specifically, we asked the participants (a) how exhausting they had found the exercise (1 = very little exhausting; 7 = very exhausting) and how comfortable they had found (b) the intensity (1 = very uncomfortable; 10 = very comfortable), (c) the duration (1 = very uncomfortable; 10 = very comfortable), and (d) the exercise overall (1 = very comfortable; 5 = very uncomfortable). Detailed content of the multimedia film clips can be found in Table 1.

**Table 1:** Detailed content of multimedia film clips for manipulating expectations.

Condition	Experimental 1 Shirt and Exercise-Effect Expectation	Experimental 2 Exercise-Effect Expectation	Control 1 No Expectation	Control 2 No Exercise-Effect Expectation
**Effects of exercise**	Current research shows that exercise has considerable positive effects on mind & body		No information about effects of exercise Information about scientific study of brain activity during exercise, the EEG method, and the EEG application in this study	Current research casts doubt on the overall benefits of exercise
	Duration and intensity of the exercise in this study are particularly suited to maximizing health benefits			Only intensive and sustained exercise results in health benefits Moderate, short exercises like in this study barely result in benefits
	Exercise will augment the secretion of happy hormones, which results in a considerable mood increase			Exercise will barely augment the secretion of happy hormones and therefore not affect mood
	Exercise will result in a relaxation of the central nervous system and a related effect on inner balance and calmness			Such an exercise is not appropriate for relaxing the central nervous system
	Exercise will reduce stress hormones, which results in improved tolerance of daily stressors			Only longer and more intense exercises will reduce stress hormones and result in more inner balance and calmness
	Although not perceivable, exercise will reduce blood pressure and decrease augmented blood sugar and fat			Bodily processes will rarely be affected by such an exercise
**Effects of compression shirt**	Due to its compression properties, the shirt supports the cardio-vascular system, thereby easing breathing during the exercise	The shirt supports removal of heat and sweat from the body for accurate EEG measurements		
	Thus, the shirt considerably intensifies the already described health effects of exercise			

In order to avoid bias, we used a double-blind design to assign participants to the various experimental and control groups (multimedia film clips). To maintain naivety in participants regarding the main research question and the respective group assignments, we provided them incorrect information about the study aim (investigation of neuronal processes during physical activity and their relation to different psychological and physiological measures) and the role of the film clips. To ensure that laboratory investigators were unable to identify group assignments of participants, we prevented laboratory investigators from seeing and hearing the multimedia film clips during the experiment (e.g., all participants wore headphones during the media presentation and were seated opposite the laboratory investigators). Blinding was maintained until final data analysis.

Preliminary analyses revealed that study groups were comparable in terms of age, BMI, fitness, physical self-concept, baseline perceived exertion, and habitual expectations (*p*s > .343; see Table 2). Intercorrelations between the main study variables are summarized in Table 3. ANOVA manipulation checks to test whether the manipulation successfully influenced individuals’ exercise experience showed that experimental groups significantly differed with respect to individuals’ retrospective assessment of how exhausting the exercise was and how comfortable the exercise was with respect to its intensity (see Table 2). Descriptively, participants in the Control condition 1 found the exercise more exhausting and the intensity less comfortable than participants in the actively manipulated conditions.

**Table 2:** Description (mean, SD) of the study groups (*N* = 78).

Variables		Experimental 1 Shirt and Exercise-Effect Expectation (*n* = 19)	Experimental 2 Exercise-Effect Expectation (*n* = 20)	Control 1 No Expectation (*n* = 20)	Control 2 No Exercise-Effect Expectation (*n* = 19)	*p*
	**Age (years)**	22.05 ± 3.29	22.60 ± 2.70	21.40 ± 3.19	21.37 ± 2.22	.486
	**Body Mass Index (kg/m**^**2**^**)**	21.82 ± 2.30	21.62 ± 2.80	21.99 ± 2.68	21.91 ± 2.88	.974
	**Physical Fitness (P**_**max**_**/kg)**	3.09 ± 0.61	3.35 ± 0.55	3.18 ± 0.63	3.17 ± 0.61	.568
	**Habitual Expectation**	6.89 ± 2.11	7.79 ± 1.65	6.79 ± 2.15	6.84 ± 2.19	.343
	**Physical Self-Concept**	3.84 ± 0.58	3.68 ± 0.73	3.62 ± 0.71	3.55 ± 0.83	.653
	**Perceived Exertion (Baseline)**	11.16 ± 1.57	11.65 ± 1.66	11.65 ± 1.57	11.42 ± 1.54	.737
**Manipulation checks**						
	**Subjective assessment of effort**	3.16 ± 1.02	3.45 ± 1.40	4.20 ± 1.20	3.26 ± 1.33	.045
	**Subjective assessment of intensity**	8.26 ± 1.52	7.30 ± 1.95	6.45 ± 1.73	7.84 ± 1.68	.011
	**Subjective assessment of duration**	7.21 ± 2.35	6.30 ± 2.32	5.65 ± 2.28	6.47 ± 2.34	.225
	**Subjective overall assessment**	2.11 ± 0.94	2.45 ± 0.95	2.70 ± 0.73	2.37 ± 0.83	.205
**Dependent variable**						
	**Perceived Exertion**	12.75 ± 0.92	13.02 ± 1.52	13.88 ± 1.44	13.16 ± 0.98	.040

*P*-values refer to between-group comparisons using analyses of variance (ANOVAs). Subjective assessment of effort: Participants rated how exhausting they found the exercise (1 = very little exhausting; 7 = very exhausting). Subjective assessment of intensity: Participants rated how comfortable they found the intensity of the exercise (1 = very uncomfortable; 10 = very comfortable). Subjective assessment of duration: Participants rated how comfortable they found the duration of the exercise (1 = very uncomfortable; 10 = very comfortable). Subjective overall assessment: Participants rated how comfortable they found the exercise overall (1 = very comfortable; 5 = very uncomfortable).

**Table 3:** Bivariate correlations for main study variables (*N* = 78).

Variables		1	2	3	4	5	6	7	8	9	10
**1.**	**Perceived Exertion**	—									
**2.**	**Age**	-.28*	—								
**3.**	**BMI**	-.12	-.08	—							
**4.**	**Physical Fitness**	.05	-.07	-.21^†^							
**5.**	**Perceived Exertion (Baseline)**	.16	-.10	.12	.18	—					
**6.**	**Habitual Expectation**	-.23*	.17	.06	.14	.01	—				
**7.**	**Contrast 1**	-.27*	.19	-.04	.05	-.10	.12	—			
**8.**	**Contrast 2**	-.15	.16	-.02	.06	-.05	.12	.78***	—		
**9.**	**Contrast 3**	-.11	-.13	.05	-.16	-.11	-.14	-.02	-.02	—	
**10.**	**Physical Self-Concept**	-.11	-.05	-.18	.33**	.08	.38**	.09	.12	.09	—

Bivariate correlations represent Spearman’s rank correlation due to non-normality of most of the data. Contrast 1 = Experimental 1 and 2 (*Shirt and Exercise-Effect Expectation/Exercise-Effect Expectati*on) vs. Control 1 (*No Expectation*). Contrast 2 = Experimental 1 and 2 vs. Control 2 (*No Exercise-Effect Expectation*). Contrast 3 = Experimental 1 (*Shirt and Exercise-Effect Expectation*) vs. Experimental 2 (*Exercise-Effect Expectation*).

### Fitness test and acute exercise in the experiment

Participants completed both the initial fitness assessment and the exercise in the main experiment on the same bicycle ergometer. Before each test, participants were instructed to adjust the saddle height for their comfort and to keep the cadence during the tests at approximately 75 pedal rotations per minute (RPM). In the initial fitness assessment, we used a standardized incremental exercise test: Participants started at 60 watts and continued with 20-watt increases every minute until volitional exhaustion. Maximal power output (P_max_) at volitional exhaustion relative to body weight was used as a measure for physical fitness (for financial reasons, as a substitute for a spiroergometry with lactate assessment). On the basis of the general linear relationship between oxygen uptake (VO_2_) and power output on a bicycle ergometer [28], P_max_ may be considered to be a valid and reliable estimate for VO_2 max_ [29]. For the main experiment, we chose a 30-minute exercise session with moderate intensity, because previous research suggests that in such circumstances psychological influences such as expectations may be most influential in affecting exercise experience in terms of perceived exertion or affect [30,31]. Moderate intensity was set to 40% of P_max_ of participants’ individual fitness test results. We determined this level as a level just below lactate threshold on the basis of a comprehensive laboratory database with 2958 fitness assessments from trained and untrained individuals (Radlabor GmbH, Freiburg, Germany) showing that the 95% confidence interval of the lactate threshold ranged from 41.1% to 65.1% (on average, 53.1%). During the main experiment, in line with previous research [32], we assessed the rate of perceived exertion (RPE; [23,24]) every five minutes.

### Measures

#### Perceived exertion

The RPE scale [23,24] measures the degree of subjective perception of physical exertion during exercise. It ranges from 6, “no exertion at all,” to 20, “maximal exertion.” Attached to the odd numbered categories of the RPE scale are verbal anchors (e.g., 11 = fairly light, 13 = somewhat hard, 15 = hard, 17 = very hard) that indicate the degree of perceived exertion. This widely used scale has been demonstrated to have adequate reliability and validity in adults [4]. Moreover, ratings of perceived exertion are strongly related to objective measurements such as heart rate and work load [33,34]. All participants were familiarized with the use of the RPE scale and indicated confidence with the use of the RPE scale prior to the experiment. For all analyses, we averaged perceived exertion ratings across time per participant.

#### Physical self-concept

We measured participants’ physical self-concept using the *physical efficiency* subscale of the Frankfurt Body Concept Scales [18]. This subscale assesses the evaluation of an individual’s physical strength and robustness as well as his or her perceived mobility, motor skills, and looseness using 10 items (e.g., “I am good at sports”) with a 6-point Likert scale ranging from 1 (strongly agree) to 6 (strongly disagree). We reversed the scale so that higher final scores indicate a higher degree of physical self-concept. In a similar sample of young adults, the Frankfurt Body Concept Scales showed satisfactory internal consistency, retest reliability, and concurrent validity [18]. In this study, internal consistency (Cronbach’s α) was .78. The questionnaire we used is included in S2 Text, both in the original language (German) and in an English translation.

#### Control variables

To control for individual differences in the evaluation of perceived exertion, we used the participants’ levels of perceived exertion at moderate intensity (40% of P_max_) from the initial fitness assessment as a baseline measure. To control for pre-existing (habitual) exercise outcome expectations, we focused specifically on mood changes, as they are among the most salient consequences of acute exercise [35] and are linked to experienced exercise intensity [36]. Therefore, we calculated an index based on the following two items from the validated *measure of beliefs about improvements in mood associated with exercise* by O'Halloran and colleagues [22]: (1) “During a run, I experience feelings of improved mood” and (2) “Following a run, I experience feelings of improved mood” (scale: 1 = not at all to 5 = totally). We selected these two items because they refer directly to the experiences of the individual participants.

### Statistical analyses

First, to examine the influence of the expectation manipulation on perceived exertion on a global level we performed a one-way analysis of variance (ANOVA) and an analysis of covariance (ANCOVA) controlling for baseline scores of perceived exertion and habitual expectations. Then we addressed specific hypotheses with hierarchical regression analyses, as they allowed us to both examine specific group-related hypotheses with regard to the expectation manipulation (similar to a set of planned contrasts in an ANOVA context) and test for an interaction between the grouping factor induced expectation and the continuous variable physical self-concept [37,38]. In these hierarchical regression analyses, we again controlled for the variables baseline perceived exertion and habitual expectation in Step 1. In Step 2 we included a set of three planned contrasts to address group-specific hypotheses regarding induced expectations, referred to in the following as Contrast 1, Contrast 2, and Contrast 3: Contrast 1 compared experimental conditions 1 and 2 (*Shirt and Exercise-Effect Expectation* and *Exercise-Effect Expectation*) versus control condition 1 (*No Expectation*), while Contrast 2 compared experimental conditions 1 and 2 versus control condition 2 (*No Exercise-Effect Expectation*). For both of these contrasts, positive values were for individuals with more positive expectation conditions. The third contrast, Contrast 3, compared experimental condition 1 with experimental condition 2 (*Shirt and Exercise-Effect Expectation* versus *Exercise-Effect Expectation*), with positive values for individuals in the former group. To test for a potentially moderating role of physical self-concept, we included physical self-concept as a predictor in Step 3 and the interaction terms for the interaction between induced expectation and physical self-concept in Step 4. To further break down significant interaction effects, we conducted contrast-specific simple slope analyses at the moderator levels (physical self-concept) *mean—1 SD*, *mean*, and *mean + 1 SD* [37] using the software PROCESS [39]. Low, medium, or high physical self-concepts refer to the values *mean—1 SD*, *mean*, and *mean + 1 SD*.

Because case-wise diagnostics indicated two potential outliers (based on standardized residuals greater than ± 2.5; [40]) in all regression analyses and because interaction effects are very susceptible to outlier effects [37], we report hierarchical regression analyses and subsequent simple slope analyses for the sample without potential outliers (*N* = 76; results for the full sample [*N* = 78] are reported in S1 Text). Although the reasons for these outliers remain speculative, inspection showed that these individuals reported substantially less perceived exertion (mean RPEs of 9.67 and 10.83, respectively) than most other individuals (mean RPE: 13.29), despite comparable physical strain. Accordingly, these individuals could have misinterpreted the RPE scale.

## Results

### Influence of expectations on perceived exertion

On a global level, participants’ perceived exertion differed significantly depending on the induced outcome expectation, $F(3,74)=2.90,p=.040,\eta_{p}^{2}=.105$ (Table 2), and still tended to differ by marginal significance after we adjusted for baseline perceived exertion and habitual expectation scores, $F(3,72)=2.54,p=.063,\eta_{p}^{2}=.096$. Results with respect to the specific groups can be found in Table 4. As expected, participants with induced positive expectations reported significantly less perceived exertion than participants in the *No Expectation* control condition (Contrast 1). However, unexpectedly, participants with induced positive expectations did not significantly differ in their perceived exertion levels from participants in the *No Exercise-Effect Expectation* control condition (Contrast 2). Furthermore, participants in positive expectation conditions did not show different perceived exertion either (Contrast 3). That is, perceived exertion did not differ among those expecting benefits from the exercise and those expecting even greater exercise benefits with additional support from the compression shirt.

**Table 4:** Hierarchical multiple regression analyses predicting perceived exertion during exercise (*N* = 76).

	Perceived Exertion		
Predictor	Δ*R*^2^	Δ*f*^2^	*β*
**Step 1**	.12*	0.14	
** Baseline Perceived Exertion**			.22^†^
** Habitual Expectation**			-.28*
**Step 2**	.13*	0.17	
** Contrast 1**			-.35**
** Contrast 2**			.08
** Contrast 3**			-.15
**Step 3**	.00	0.00	
** Physical Self-Concept**			-.07
**Step 4**	.16**	0.27	
** Physical Self-Concept ×** **Contrast 1**			-.04
** Physical Self-Concept ×** **Contrast 2**			-.24*
** Physical Self-Concept ×** **Contrast 3**			.31**
**Total *R***^**2**^	.41***		

Contrast 1 = Experimental 1 and 2 (*Shirt and Exercise-Effect Expectation*/*Exercise-Effect Expectation*) vs. Control 1 (*No Expectation*). Contrast 2 = Experimental 1 and 2 vs. Control 2 (*No Exercise-Effect Expectation*). Contrast 3 = Experimental 1 (*Shirt and Exercise-Effect Expectation*) vs. Experimental 2 (*Exercise-Effect Expectation*). **Δ** Cohen’s f^2^ represents the individual contribution of the additional predictor/set of predictors. According to Cohen [41], effect sizes f^2^ of 0.02, 0.15, and 0.35 are considered small, medium, and large, respectively.

^†^ *p* < .10.

* *p* < .05.

** *p* < .01.

*** *p* < .001.

### The moderating role of physical self-concept

**Fig 2:**
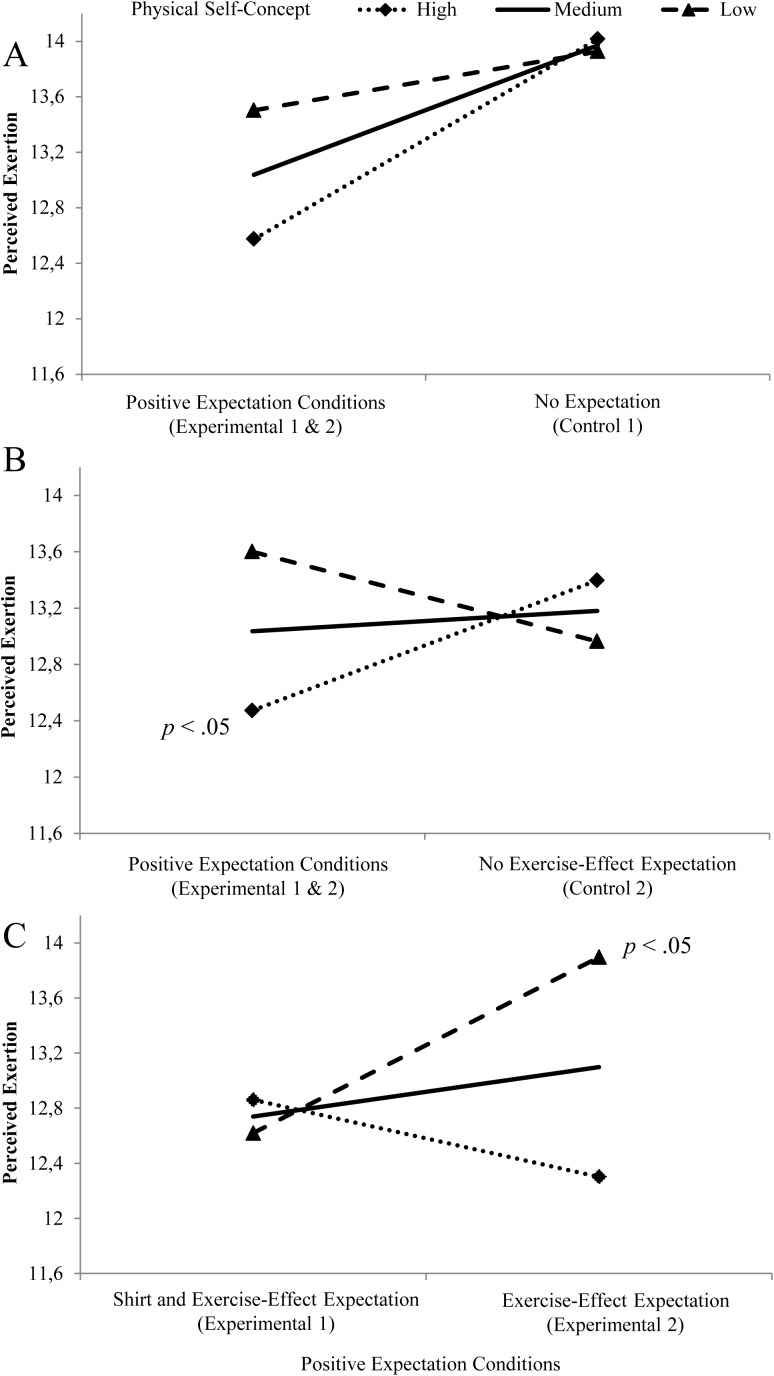
Visual representation of interaction effects between expectation-related contrasts and physical self-concept on perceived exertion. Conditional effects of (A) experimental conditions 1 and 2 (*induced positive expectations*) versus Control 1 (Contrast 1), (B) experimental conditions 1 and 2 (*induced positive expectations*) versus Control 2 (Contrast 2) and (C) Experimental 1 (*Shirt and Exercise-Effect Expectation*) versus Experimental 2 (*Exercise-Effect Expectation*) (Contrast 3) on perceived exertion among participants with high (mean + 1 SD), medium (mean), and low physical self-concept (mean—1 SD). Endpoints represent estimated means of perceived exertion for these moderator levels, controlled for habitual expectation and baseline perceived exertion. Significant simple slopes are indicated (for [B] on the level of high physical self-concept, for [C] on the level of low physical self-concept). All figures are based on *N* = 76.

With respect to the interaction between outcome expectations and physical self-concept, we expected increased effects of positive expectations in participants with a high physical self-concept. Regression analyses did not reveal an effect for the interaction between induced expectations (Contrast 1) and physical self-concept (see Table 4: Step 4 and Fig 2A). However, as expected, regression analyses yielded a significant effect for the interaction between induced expectations (Contrast 2) and physical self-concept. To further decompose the interaction effect, we conducted simple slope analyses (*N* = 57). They showed that participants with induced positive expectations only reported reduced perceived exertion compared to participants in control condition 2 (*No Exercise-Effect Expectation*) if they had a high physical self-concept (*b* = 0.308, *p* = .024, 95% CI [0.04, 0.57]). For individuals with medium (*b* = 0.048, *p* = .603, 95% CI [-0.14, 0.23]) or low physical self-concepts (*b* = -0.212, *p* = .098, 95% CI [-0.47, 0.04]), perceived exertion levels did not differ between induced positive expectation conditions and control condition 2 (*No Exercise-Effect Expectation*). Fig 2B visualizes this interaction effect.

For participants with a low physical self-concept, we expected positive expectations related to an external supportive product to be most effective in reducing perceived exertion. To examine this issue, we tested the interaction between physical self-concept and induced expectations (Contrast 3), that is, *Shirt and Exercise-Effect Expectation* vs. *Exercise-Effect Expectation*. This interaction significantly predicted perceived exertion (Table 4: Step 4). Further simple slope analyses (*N* = 38) showed that participants with a low physical self-concept reported reduced levels of perceived exertion during the exercise when they additionally believed in the enhancing qualities of the compression shirt (*b* = 1.277, *p* = .020, 95% CI [0.22, 2.33]). For participants with a medium (*b* = 0.359, *p* = .285, 95% CI [-0.31, 1.03]) or high physical self-concept (*b* = -0.559, *p* = .217, 95% CI [-1.46, 0.35]), in contrast, an additional shirt-related expectation did not significantly affect perceived exertion levels. Fig 2C visualizes this interaction effect.

## Discussion

The main purpose of the present study was to examine how placebo expectations influence perceived exertion during exercise. Our first hypothesis, namely that placebo expectations predict perceived exertion during exercise, is partially supported by our results: Participants with positive outcome expectations showed lower perceived exertion during the activity on the ergometer than those without specific expectations in the Control 1 condition. In considering the Control 2 condition, however, we also found conflicting evidence that warrants further investigation. With respect to our second hypothesis, the results indicate that physical self-concept serves as a moderator: Participants with a high physical self-concept benefited particularly in terms of reduced perceived exertion levels from an induction of generally positive expectations compared to the induction of a *No-Exercise Effect Expectation* (Control 2). However, perceived exertion did not differ among those only expecting exercise benefits and those additionally expecting equipment benefits. In contrast, results regarding our third hypothesis revealed that only participants with a low physical self-concept were able to benefit in terms of their perceived exertion when additional attention was drawn to supposedly enhancing properties of the exercise equipment (compression shirt). The implications of these findings are discussed below.

### Influence of expectations on perceived exertion

On the one hand, by showing that participants with generally positive outcome expectations reported less perceived exertion than participants without a particular outcome expectation (Control 1), our research suggests that individuals’ outcome expectations affect their perceptions of exertion during exercise. The suggested role of outcome expectations in perceived exertion is in line with previous research indicating that several other psychological variables influence exertional experience [8]. In particular, our results are consistent with results demonstrating that different cognitive modes affect perceived exertion: For instance, people report reduced perceived exertion when they listen to music and watch associated music videos while exercising [42]. Similarly, individuals report increased perceived exertion when they believe that they have nearly completed their exercise [43] and when they believe that they are cycling up a steep hill (expectations induced by hypnotic suggestion) [44].

On the other hand, however, and contradictory to our expectations, we did not observe differences in perceived exertion among participants expecting positive outcomes and those not expecting positive outcomes (Control 2). This discrepant finding clearly limits the interpretability of the aforementioned main effect. Indeed, one potential reason for these discrepant findings may well be that differences in perceived exertion found between the positive expectation and Control 1 conditions are the result of an artifact, that is, that a main effect of positive outcome expectations on perceived exertion does not exist. For instance, it is conceivable that the film clip in the Control 1 condition, which included more vivid information regarding the study, may have acutely resulted in more negative emotions such as anxiety, which in turn contributed to higher levels of perceived exertion [45]. In contrast, a second potential reason for the discrepancy may be that there still is a main effect of positive outcome expectations but this was not adequately captured with the Control 2 condition. This might have been the case, for instance, if the film clip in the Control 2 condition was not as effective as we intended. Our participants might not have fully believed in the story presented in the Control 2 condition (the present exercise is not long and vigorous enough to result in acute benefits) due to its counterintuitive message. Instead, the discussion of exercise health effects in the film clip may have primed the participants to believe in beneficial exercise aspects in the first place. In fact, on the basis of the perceived exertion and retrospective manipulation check data, it seems that participants in the Control 2 condition experienced the exercise similarly to the participants in positive expectation conditions. Unfortunately, because we did not collect further data on the acute effects of the film clips, such as subsequent expectations and emotions, the discrepancy between these two findings cannot be resolved in this study. Therefore, a potential effect of positive outcome expectations on perceived exertion must be interpreted with caution.

In order to rule out potential alternative explanations (e.g., emotions elicited by the film clip instead of outcome expectations influenced perceived exertion during the exercise [45]), it will be necessary to conduct further experiments manipulating outcome expectations experimentally while controlling for emotions caused by the experimental manipulation [46]. Future research in this field would also benefit from an assessment of participants’ expectation directly after the experimental manipulation when one can assume that this assessment does not draw their attention to the true aim of the study [46, 47]. In addition, it would be helpful at the end of future experiments to include both suspicion checks to determine whether participants were skeptical about the cover story of the experiment and items assessing participants’ knowledge of placebo effects in order to additionally control for the influence of participants’ understanding of mind–body effects [46].

### The moderating role of physical self-concept

This study indicated that physical self-concept plays a moderating role: Particularly for participants with a high physical self-concept, positive expectation induction resulted in reduced perceived exertion compared to the induction of the *No Exercise-Effect Expectation* (Control 2). This is consistent with the general notion that individuals with certain personality characteristics are more prone to placebo responses than others [48], and more specifically, it is in line with findings showing that placebo interventions are more effective when individuals think positively about their own abilities [16]. Accordingly, physical self-concept may be an important variable to consider when one wants to take advantage of the role of expectations to reduce exertion levels in exercise contexts.

Our results also indicate that only participants who evaluated their physical abilities as being low (low physical self-concept) reported reduced perceived exertion when they were primed on potential positive aspects of the exercise product they were wearing (compression shirt). These results are in line with previous findings from marketing research suggesting that product-related beliefs can have a powerful effect on the actual effectiveness of a particular product, such as the compression shirt worn by the participants in our study (e.g., [14]). Moreover, similar to the findings of Garvey and colleagues [17], our results suggest that individuals with rather negative attitudes regarding their own physical abilities derive particular benefit from positive expectations with respect to the beneficial qualities of an exercise product.

### Unresolved issues

Several questions are left unanswered. First, how did positive expectations influence exertional experience? For instance, one potential psychological mechanism may be that individuals’ anticipation of exercise benefits resulted in increased attention towards body symptoms [49], leading them to identify more positive expectation-congruent sensations than expectation-incongruent exertional symptoms [12,50]. As identifying potential mechanisms of action was beyond the scope of the current study, future research should examine how expectations influence attentional processes and attributions of exertional symptoms. Second, what are the limitations of these expectation effects? We used a standardized moderate-intensity exercise to examine effects of expectations. However, by using a percentage (40%) of maximal power output relative to body weight instead of a more direct measure of aerobic/anaerobic metabolism at different exercise levels to standardize exercise intensity, we may have over- or underestimated intensity levels for certain individuals. Therefore, this study is limited with regard to the evaluation of expectation effects on perceived exertion at a specific intensity level. Nevertheless, previous research also suggests that cognitive influence on exercise experience, such as that of placebo expectations, may be largest at moderate intensities (dual mode theory, [30]). Whether and how the effect of expectations on exertion may be generalized across different intensities has yet to be explored. Third, the generalizability across samples also remains open. This study used a sample of young and healthy sedentary individuals. Thus, future research should further investigate clinical samples as well as active and older individuals.

### Implications of the current findings

Despite these limiting aspects, our research has several implications. First, our approach provides insight into a new class of factors that potentially influence perceived exertion during exercise. Moreover, by indicating how expectations influence perceived exertion, these findings may have practical relevance for health-oriented exercisers. For instance, knowing about the role of expectations may help them to improve their exercise experience and long-term exercise adherence. Our research may also have implications for the marketing of exercise and sports equipment. The results imply that people with negative attitudes about their physical abilities in particular may “get what they pay for” when consuming sports products such as compression shirts. For instance, while there is only little evidence that the use of compression garments results in advantages for performance or well-being in general [51, 52], individuals with negative physical self-concepts may benefit from their use in the manner of a self-fulfilling prophecy. Last, our findings have more general implications for placebo research as they unravel another aspect of everyday life—exertional experience during exercise—in which the well-studied placebo-effect may play an important role.

## Supporting information

S1 Dataset (XLSX)

S1 Text Results of hierarchical regression analyses and simple slope analyses for the full sample (*N* = 78). (DOCX)

S2 Text Additional information on used survey questions and questionnaires. Survey questions and questionnaires used in the study both in the original language (German) and in an English translation.
(DOCX)
