# Supplementary material for: Do placebo expectations influence perceived exertion during physical exercise?
Source: PLoS One. 2017 Jun 29;12(6):e0180434. doi: 10.1371/journal.pone.0180434 (PMC5491246; doi:10.1371/journal.pone.0180434)
Supplement: S1 Text — (DOCX) [file pone.0180434.s002.docx]

**S1 Text. Results of hierarchical regression analyses and simple slope analyses for the full sample (*N*=78).**

**Table A. Hierarchical multiple regression analyses predicting perceived exertion during exercise using the full sample (N = 78).**

|  | **Perceived Exertion** | | |
| --- | --- | --- | --- |
| **Predictor** | **∆*R*^2^** | **∆*f*^2^** | ***β*** |
| **Step 1** | .10* | 0.11 |  |
| **Baseline Perceived Exertion** |  |  | .19^†^ |
| **Habitual Expectation** |  |  | -.27* |
| **Step 2** | .09^†1^ | 0.11 |  |
| **Expectation Contrast 1** |  |  | -.29* |
| **Expectation Contrast 2** |  |  | .04 |
| **Expectation Contrast 3** |  |  | -.10 |
| **Step 3** | .00 | 0.00 |  |
| **Physical Self-Concept** |  |  | -.06 |
| **Step 4** | .14** | 0.21 |  |
| **Physical Self-Concept ×**  **Expectation Contrast 1** |  |  | -.05 |
| **Physical Self-Concept ×**  **Expectation Contrast 2** |  |  | -.21^†2^ |
| **Physical Self-Concept ×**  **Expectation Contrast 3** |  |  | .29** |
| **Total *R*^2^** | .33** |  |  |

Expectation Contrast 1 = *Shirt and Exercise-Effect Expectation/Exercise-Effect Expectati*on (Experimental 1 and 2) vs. *No Expectation* (Control 1). Expectation Contrast 2 = *Shirt and Exercise-Effect Expectation/Exercise-Effect Expectation* (Experimental 1 and 2) vs. *No Exercise-Effect Expectation* (Control 2). Expectation Contrast 3 = Contrast between experimental conditions 1 and 2 (*Shirt and Exercise-Effect Expectation* vs. *Exercise-Effect Expectation*). ∆ Cohen’s f^2^ represents the individual contribution of the additional predictor/set of predictors. According to Cohen [52], effect sizes f^2^ of 0.02, 0.15, and 0.35 are considered small, medium, and large, respectively.

^†^ *p* < .10. * *p* < .05. ** *p* < .01. ^1^ *p* = .063. ^2^ *p* = .054.

**Investigation of potential interaction effects using simple slope analyses**

We conducted further simple slope analyses (*N*= 58) to decompose the potential interaction effect *Physical Self-Concept* × *Expectation Contrast 2*. The analyses revealed that participants with induced positive expectations only reported less perceived exertion than participants in control condition 2 (*No Exercise-Effect Expectation*) if they had a high physical self-concept (*b* = 0.336, *p* = .023, 95% CI [0.05, 0.62]). For individuals with medium (*b* = 0.075, *p* = .454, 95% CI [-0.12, 0.27]) or low physical self-concepts (*b* = -0.186, *p* = .179, 95% CI [-0.46, 0.09]), perceived exertion levels did not differ between induced positive expectation conditions and control condition 2 (*No Exercise-Effect Expectation*).

We also conducted further simple slope analyses (*N*=39) to decompose the interaction effect *Physical Self-Concept* × *Expectation Contrast 3*. The analyses revealed that participants with a low physical self-concept tended to report reduced levels of perceived exertion during the exercise when they additionally believed in the enhancing qualities of the compression shirt (*b* = 1.106, *p* = .071, 95% CI [-0.10, 2.31]). For participants with a medium (*b* = 0.188, *p* = .620, 95% CI [-0.58, 0.95]) or high physical self-concept (*b* = -0.730, *p* = .157, 95% CI [-1.75, 0.29]), in contrast, an additional shirt-related expectation did not affect perceived exertion levels.
