# Supplementary material for: Do placebo expectations influence perceived exertion during physical exercise?
Source: PLoS One. 2017 Jun 29;12(6):e0180434. doi: 10.1371/journal.pone.0180434 (PMC5491246; doi:10.1371/journal.pone.0180434)
Supplement: S2 Text — Survey questions and questionnaires used in the study both in the original language (German) and in an English translation. (DOCX) [file pone.0180434.s003.docx]

**S2 Text. Additional information on used survey questions and questionnaires.**

**Survey questions and questionnaires in the original language (German)**

1. **Allgemeine Angaben**
   1. **Geschlecht Männlich ( ) Weiblich ( )**
   2. **Wie alt sind Sie? ______ Jahre**
   3. **Wie groß und wie schwer sind Sie? ( ) cm ( ) kg**
2. **Körperselbstkonzept**

| **Wie sehr stimmen Sie folgenden Aussagen zu?** | **1** | **2** | **3** | **4** | **5** | **6** |
| --- | --- | --- | --- | --- | --- | --- |
|  | **Trifft sehr zu** | **Trifft zu** | **Trifft etwas zu** | **Trifft eher nicht zu** | **Trifft nicht zu** | **Trifft gar nicht zu** |
| 1. Ich bin gut im Sport. | ❑ | ❑ | ❑ | ❑ | ❑ | ❑ |
| 1. Ich bin motorisch sehr ungeschickt. | ❑ | ❑ | ❑ | ❑ | ❑ | ❑ |
| 1. Ich empfinde mich als ausgesprochen steif. | ❑ | ❑ | ❑ | ❑ | ❑ | ❑ |
| 1. Ich habe oft das Gefühl, dass mein Gang steif und hölzern wirkt. | ❑ | ❑ | ❑ | ❑ | ❑ | ❑ |
| 1. Ich bin ein sportlicher Typ. | ❑ | ❑ | ❑ | ❑ | ❑ | ❑ |
| 1. Ich bin stark. | ❑ | ❑ | ❑ | ❑ | ❑ | ❑ |
| 1. Ich bin froh über meine körperliche Zähigkeit. | ❑ | ❑ | ❑ | ❑ | ❑ | ❑ |
| 1. Ich treibe viel Sport. | ❑ | ❑ | ❑ | ❑ | ❑ | ❑ |
| 1. Die Schwerfälligkeit meiner Bewegungen ärgert mich. | ❑ | ❑ | ❑ | ❑ | ❑ | ❑ |
| 1. Ich bin allgemein sehr verkrampft. | ❑ | ❑ | ❑ | ❑ | ❑ | ❑ |

1. **Habituelle Erwartungshaltungen**

| **Wie sehr stimmen Sie folgenden Aussagen zu?** | **Trifft nicht oder fast nicht zu** | **Trifft eher nicht zu** | **Trifft teilweise zu** | **Trifft eher zu** | **Trifft genau zu** |
| --- | --- | --- | --- | --- | --- |
| 1. Ich bemerke Stimmungsverbesserungen bei mir, **während** ich Sport treibe. | ❑ | ❑ | ❑ | ❑ | ❑ |
| 2. Ich bemerke Stimmungsverbesserungen bei mir, **nachdem** ich Sport getrieben habe. | ❑ | ❑ | ❑ | ❑ | ❑ |

1. **Wahrgenommenes Anstrengungserleben**

| **Schätzung des Anstregungsempfindens** | | | |
| --- | --- | --- | --- |
| **Skala** | **Beschreibung** | | **Beispiel** |
| 6 | Überhaupt nicht anstrengend | | Aktivitäten wie Liegen oder Stehen |
| 7 | Extrem leicht | |  |
| 8 |  |  |  |
| 9 | Sehr leicht | | Normales Gehen im eigenen Tempo |
| 10 |  | |  |
| 11 | Leicht | |  |
| 12 |  | |  |
| 13 | Etwas anstrengend | | Man kann bei der Belastung noch gut weitermachen |
| 14 |  | |  |
| 15 | Anstrengend | schwer | Fortfahren ist noch möglich |
| 16 |  | |  |
| 17 | Sehr anstrengend | | Sie sind bald erschöpft |
| 18 |  | |  |
| 19 | Extrem anstrengend | | Stärkste Belastung, die jemals erlebt wurde |
| 20 | Maximale Anstrengung | | Anstrengung in äußerster Gefahrensituation |

1. Manipulation Check

| **Wie belastend fanden Sie die Aktivität auf dem Ergometer?** | | | | | | |
| --- | --- | --- | --- | --- | --- | --- |
| Sehr wenig belastend |  |  |  |  |  | Sehr belastend |
| ❑  1 | ❑  2 | ❑  3 | ❑  4 | ❑  5 | ❑  6 | ❑  7 |

| **Wie angenehm war für Sie die Intensität der Aktivität auf dem Ergometer?** | | | | | | | | | | |
| --- | --- | --- | --- | --- | --- | --- | --- | --- | --- | --- |
| Sehr unangenehm | | | | | Sehr angenehm | | | | | |
| ❑  1 | ❑  2 | ❑  3 | ❑  4 | ❑  5 | | ❑  6 | ❑  7 | ❑  8 | ❑  9 | ❑  10 |

| **Wie angenehm war für Sie die Dauer der Aktivität auf dem Ergometer?** | | | | | | | | | | |
| --- | --- | --- | --- | --- | --- | --- | --- | --- | --- | --- |
| Sehr unangenehm | | | | | | Sehr angenehm | | | | |
| ❑  1 | ❑  2 | ❑  3 | ❑  4 | ❑  5 | ❑  6 | | ❑  7 | ❑  8 | ❑  9 | ❑  10 |

| **Wie angenehm war für Sie die Aktivität insgesamt?** | | | | |
| --- | --- | --- | --- | --- |
| Sehr angenehm | Angenehm | Weder… noch… | Unangenehm | Sehr unangenehm |
| ❑ | ❑ | ❑ | ❑ | ❑ |

**Survey questions and questionnaires in an English translation**

1. **General information**
   1. **Sex Male ( ) Female ( )**
   2. **How old are you? ______ years**
   3. **How tall are you and how much do you weigh? ( ) cm ( ) kg**
2. **Physical self-concept**

| **To what extent do you agree or disagree with each of these statements?** | **1** | **2** | **3** | **4** | **5** | **6** |
| --- | --- | --- | --- | --- | --- | --- |
|  | **Strongly agree** | **Agree** | **Some-what agree** | **Some-what disagree** | **Disagree** | **Strongly disagree** |
| 1. I am good at sports. | ❑ | ❑ | ❑ | ❑ | ❑ | ❑ |
| 1. I have very poor motor skills. | ❑ | ❑ | ❑ | ❑ | ❑ | ❑ |
| 1. I see myself as markedly stiff. | ❑ | ❑ | ❑ | ❑ | ❑ | ❑ |
| 1. I often have the feeling that my gait appears stiff and clunky. | ❑ | ❑ | ❑ | ❑ | ❑ | ❑ |
| 1. I am a sporty type. | ❑ | ❑ | ❑ | ❑ | ❑ | ❑ |
| 1. I am strong. | ❑ | ❑ | ❑ | ❑ | ❑ | ❑ |
| 1. I am glad about my physical toughness. | ❑ | ❑ | ❑ | ❑ | ❑ | ❑ |
| 1. I work out a lot. | ❑ | ❑ | ❑ | ❑ | ❑ | ❑ |
| 1. The clunkiness of my movements annoys me. | ❑ | ❑ | ❑ | ❑ | ❑ | ❑ |
| 1. I am very cramped in general. | ❑ | ❑ | ❑ | ❑ | ❑ | ❑ |

1. **Habitual Expectation**

| **To what extent do you agree or disagree with each of these statements?** | **Never or almost never agree** | **Some-what disagree** | **Partially agree** | **Some-what agree** | **Always agree** |
| --- | --- | --- | --- | --- | --- |
| 1. **During** a run, I experience feelings of improved mood. | ❑ | ❑ | ❑ | ❑ | ❑ |
| 2. **Following** a run, I experience feelings of improved mood. | ❑ | ❑ | ❑ | ❑ | ❑ |

1. **Perceived Exertion**

| **Estimation of perceived exertion** | | | |
| --- | --- | --- | --- |
| **Scale** | **Description** | | **Example** |
| 6 | No exertion at all | | Activities such as lying or standing |
| 7 | Extremely light | |  |
| 8 |  |  |  |
| 9 | Very light | | Normal walking at your own pace |
| 10 |  | |  |
| 11 | Light | |  |
| 12 |  | |  |
| 13 | Somewhat hard | | It feels okay to continue |
| 14 |  | |  |
| 15 | Hard | heavy | Continuing is still possible |
| 16 |  | |  |
| 17 | Very hard | | You are exhausted soon |
| 18 |  | |  |
| 19 | Extremely hard | | Strongest exertion you have ever experienced |
| 20 | Maximal exertion | | Exertion in most extreme dangerous situation |

1. Manipulation Check

| **How exhausting did you find the exercise on the ergometer?** | | | | | | |
| --- | --- | --- | --- | --- | --- | --- |
| Very little exhausting |  |  |  |  |  | Very exhausting |
| ❑  1 | ❑  2 | ❑  3 | ❑  4 | ❑  5 | ❑  6 | ❑  7 |

| **How comfortable did you find the intensity of the exercise on the ergometer?** | | | | | | | | | | |
| --- | --- | --- | --- | --- | --- | --- | --- | --- | --- | --- |
| Very uncomfortable | | | | | Very comfortable | | | | | |
| ❑  1 | ❑  2 | ❑  3 | ❑  4 | ❑  5 | | ❑  6 | ❑  7 | ❑  8 | ❑  9 | ❑  10 |

| **How comfortable did you find the duration of the exercise?** | | | | | | | | | | |
| --- | --- | --- | --- | --- | --- | --- | --- | --- | --- | --- |
| Very uncomfortable | | | | | | Very comfortable | | | | |
| ❑  1 | ❑  2 | ❑  3 | ❑  4 | ❑  5 | ❑  6 | | ❑  7 | ❑  8 | ❑  9 | ❑  10 |

| **How comfortable did you find the exercise overall?** | | | | |
| --- | --- | --- | --- | --- |
| Very comfortable | Comfortable | Neutral | Uncomfortable | Very uncomfortable |
| ❑ | ❑ | ❑ | ❑ | ❑ |
